# Effect of intra-pregnancy nonsurgical periodontal therapy on inflammatory biomarkers and adverse pregnancy outcomes: a systematic review with meta-analysis

**DOI:** 10.1186/s13643-017-0587-3

**Published:** 2017-10-10

**Authors:** Helbert Eustáquio Cardoso da Silva, Cristine Miron Stefani, Nilce de Santos Melo, Adriano de Almeida de Lima, Cassiano Kuchenbecker Rösing, André Luís Porporatti, Graziela De Luca Canto

**Affiliations:** 1 Post-Graduation Program, Foundation for Teaching and Research in Health Sciences, Brasilia, Brazil; 2 Dentistry Department, Faculty of Health Science, Brasilia University, Brasilia, Brazil; 3 Department of Conservative Dentistry, Faculty of Dentistry, Federal University of Rio Grande do Sul, Porto Alegre, Brazil; 4 Brazilian Centre for Evidence-based Research, Department of Dentistry, Federal University of Santa Catarina, Florianopolis, Santa Catarina Brazil; 5 School of Dentistry, Faculty of Medicine and Dentistry University of Alberta, Edmonton, Canada; 6 Condomínio Mansões Entrelagos, Etapa 1, conjunto S, casa 9, Paranoá, Brasília, DF 73255-900 Brazil

**Keywords:** Nonsurgical periodontal therapy, Pregnancy, Women’s health, Cytokine(s), Systematic review, Meta-analysis

## Abstract

**Background:**

The aim of this systematic review with meta-analysis was to analyze the effects of intra-pregnancy nonsurgical periodontal therapy on periodontal inflammatory biomarkers and adverse pregnancy outcomes.

**Methods:**

On June 5, 2017, we searched PubMed, Cochrane, SCOPUS, Web of Science, LILACS, ProQuest, Open Grey, and Google Scholar databases. Randomized clinical trials in which pregnant women with chronic periodontitis underwent nonsurgical periodontal therapy, compared with an untreated group, tested for inflammatory biomarkers, and followed till delivery were included. Primary outcomes were preterm birth, low birth weight, and preeclampsia. Meta-analysis was performed with 5.3.5 version of Review Manager software.

**Results:**

We found 565 references in the databases, 326 after duplicates removal, 28 met criteria for full text reading, and 4 met eligibility criteria for quantitative and qualitative synthesis. Intra-pregnancy nonsurgical periodontal therapy improved periodontal clinical parameters (periodontal pocket depth, clinical attachment level, and bleeding on probing) and reduced biomarker level from gingival crevicular fluid (GCF), and some from blood serum; however, it did not influence biomarker level from umbilical cord blood. Meta-analysis showed tendency for reduction of the risk of preterm birth before 37 weeks for treated group (risk ratio (RR) = 0.54, 95% CI 0.38–0.77; *p* = 0.0007; inconsistency indexes (I2) 32%) but did not show any difference for low birth weight occurrence (RR = 0.78, 95%CI 0.50–1.21; *p* = 0.27; I2 41%). No included study considered preeclampsia as a gestational outcome.

**Conclusions:**

These results demonstrated that the intra-pregnancy nonsurgical periodontal therapy decreased periodontal inflammatory biomarker levels from gingival crevicular fluid and some from serum blood, with no influence on inflammatory biomarker level from cord blood, and it did not consistently reduce adverse gestational adverse outcome occurrence.

**Systematic review registration:**

PROSPERO CRD42015027750

**Electronic supplementary material:**

The online version of this article (10.1186/s13643-017-0587-3) contains supplementary material, which is available to authorized users.

## Background

Periodontitis is a chronic destructive inflammatory disease affecting the tooth-supporting tissues, initiated by dental plaque, a biofilm with gram-negative anaerobic microorganism’s predominance [1, 2] and mediated by the inflammatory response of the host [2–4].

Frequent episodes of bacteremia or dissemination of bacterial endotoxins from periodontal focus might induce systemic activation of the inflammatory response [5, 6] and intense pro-inflammatory cytokine production [7]. Therefore, anti-infective periodontal therapies, once reducing exposure to subgingival pathogenic microorganisms and products, might have an anti-inflammatory potential and therefore be considered an anti-inflammatory intervention [8].

The transit of periodontal pathogens, pro-inflammatory cytokines, and prostaglandins from periodontal pockets to the fetal-placental unit suggests a plausible hypothesis for the association between periodontal disease and complications of pregnancy, as premature births and low birth weight. This possibility resulted in a series of epidemiological and interventional studies, conducted in the last 20 years, to investigate the association between periodontal disease and adverse pregnancy outcomes (APO), with conflicting results [9–14]. Systematic reviews and meta-analyses suggested this diversity might be, in part, due to methodological inconsistencies which also difficult comparisons between studies. Alternative explanations include variations in the populations assessed, the presence of a range of potential confounding factors, variations in the case-definition of periodontitis across studies, relative obstetric risk, and other factors which are known to influence the prevalence of APO, irrespective of oral status [4, 15].

Measurement of inflammatory biomarkers in gingival crevicular fluid (GCF) or saliva may be used to determine the state of periodontal inflammation, which may be enhanced by periodontal therapy, reducing inflammatory biomarker level of cytokines such as IL-1β, IL-6, IL-8, IL-10, IL-12, IL-17, TNF-α, chemokines (MCP-1, RANTES), and prostaglandins in the affected sites [16]. This reduction of inflammatory biomarker levels is also associated with significant improvements in the clinical parameters of the disease, such as probing depths, percentage of bleeding on probing, and dental plaque [17].

As pregnancy progresses, amniotic fluid levels of inflammatory cytokines, such as TNF-α, IL-1β, and prostaglandin E2 (PGE2), naturally rise until a critical level, thus inducing rupture of the amniotic sac membranes, uterine contraction, cervical dilation, and delivery. Besides normal delivery is controlled by inflammatory signaling, this triggering mechanism can be modified by external stimuli, as infection and inflammatory stressors. In fact, obstetric studies associated increased levels of local and systemic markers of inflammation with APO [15].

A significantly positive linear trend was observed between the degree of periodontal disease and inflammatory mediators among preconception women. The levels of inflammatory mediators (IL-1β, IL-6, β-glucuronidase, and TNF-α) in saliva and in serum (except IL-6) significantly increased with severity of periodontal disease. The elevated concentrations of serum IL-1β, β-glucuronidase, and TNF-α-accompanying saliva mediators suggested periodontal disease might be one of the factors triggering a systemic inflammatory response [18].

In two recent studies conducted with women immediately after delivery, GCF levels of IL-1β, IL-6, TNF-α, and PGE2 and serum levels of TNF-α and PGE2 were significantly increased in women with periodontitis compared with periodontally healthy women. A significantly positive correlation was observed between periodontal disease (PD) and clinical attachment level (CAL) and serum levels of PGE2, considered the main labor trigger. A significantly positive correlation was observed between serum PGE2 and GCF levels of TNF-α. These findings might indicate that periodontal inflammation affects circulatory levels of labor triggers [19]. Severe clinical attachment loss was also associated with higher serum levels of TNF-α and IL-1β [20].

A systematic review was conducted to evaluate association between periodontal inflammatory biomarkers from GCF, as IL-1β, PGE2, and TNF-α, and APO, suggesting that a positive association between GCF inflammatory biomarker level and APO might exist [21]. Therefore, intra-pregnancy nonsurgical periodontal therapy (IPPT), once reducing periodontal inflammatory biomarker level, should interfere with APO. Some interventional studies on the effects of IPPT on APO also tested periodontal inflammatory biomarkers as inflammation resolution signs or prognostic markers to predict pregnancy outcomes [10, 13, 16, 22, 23]. However, there is no systematic review available on the potential association between biomarker changes and APO occurrence after IPPT.

The primary aim of this systematic review with meta-analysis was to analyze the effects of IPPT on periodontal inflammatory biomarkers and gestational adverse outcomes. The focused question was “In pregnant women with chronic periodontitis, does nonsurgical periodontal therapy change inflammatory biomarker level and consequently the occurrence of premature births, low birth weight or maternal preeclampsia?”

## Methods

The protocol of this study was registered on the International Prospective Register of Systematic Reviews-PROSPERO (https://www.crd.york.ac.uk/prospero/display_record.asp?ID=CRD42015027750) under number CRD42015027750. This systematic review was conducted according to the Preferred Reporting Items for Systematic Reviews and Meta-analyses, following the PRISMA checklist (http://www.prisma-statement.org/) Additional file 1.

### Search strategy

On June 5, 2017, a broad search of articles without language or time limits was performed in the following databases: PubMed, Cochrane Library, SCOPUS, Web of Science, LILACS (Latin American and Caribbean Literature), and gray literature through ProQuest, Open Grey, and Google Scholar. The Medical Subject Headings (MeSH) terms were used to develop the search strategy and to acquire the main strategy on PubMed. When words with different spelling appeared, synonyms that were in the MeSH terms were used. This strategy was adapted for the other databases. On LILACS, the same keywords in Spanish and Portuguese were used. The search strategy used is in Appendix I (see Additional file 2). Manual searches of reference lists of relevant articles, theses, and dissertations were also performed.

Immediately after literature search, the references were exported to reference manager software (ENDNOTE® X7.01, Bld 7212, Thomson Reuters) and duplicated references were removed.

### Inclusion and exclusion criteria

PICO (Population, Intervention, Comparison and Outcomes) research strategy was used to define inclusion and exclusion criteria. As inclusion criteria, randomized clinical trials in which pregnant women diagnosed with chronic periodontitis by clinical examination, underwent nonsurgical periodontal therapy (treated group) compared with those untreated (untreated group), and tested for inflammatory biomarkers and followed till delivery were selected. Preterm birth (< 37 weeks), low birth weight (≤ 2500 g), and preeclampsia (140–160 to 90–110 mm/Hg) were the primary outcomes considered.

Exclusion criteria comprised 1—reviews, editorials, letters, conferences, summaries, books, and opinions; 2—in vitro studies and studies in animal models; 3—qualitative studies; 4—cross-sectional, case-control, cohort studies, or any interventional design other than randomized clinical trials; 5—studies in which case definition of low birth weight babies included babies with weight > 2500 g or preterm births included delivery > 37 weeks; 6—studies considering any periodontal condition other than chronic periodontitis; 7—studies testing alternative/comparative periodontal treatment (antibiotics, surgery); 8—studies with no considered primary outcomes (preterm birth, low birth weight) or in which inflammatory biomarkers were not tested; 9—studies including pregnant women with co-morbidities (diabetes or gestational diabetes, heart disease, HIV, obesity, genitourinary infection); 10—studies including pregnant women with extensive dental treatment needs (presence of severe dental caries, endodontic compromised teeth and extraction needs during pregnancy).

### Data extraction

The article selection was performed in two phases. On phase 1, two independent reviewers (HECS and CMS) evaluated titles and abstracts of all articles, according to the eligibility criteria. On phase 2, both reviewers (HECS and CMS) independently read the full texts according to the inclusion and exclusion criteria. In case of disagreements, both reviewers discussed and, if consensus was not reached, a third reviewer (NSM) analyzed the articles to reach a final decision.

### Tabulation of findings

Data extraction was also performed by two independent reviewers (HECS and CMS) and posteriorly compared. Extracted data comprised author, year, country, sample, and age in years (mean ± SD and/or range); case definitions of periodontal disease (PD) and premature birth (PTB)/low birth weight (LBW); groups (*n* and treatments); inflammatory biomarkers tested and source; primary outcomes (incidence of PTB/LBW, expressed as number of cases/total deliveries per group, percent and *p* value between groups); inflammatory biomarker level differences between groups (*p* value); and main conclusions of each paper.

### Risk of bias assessment

Studies risk of bias was assessed through The Cochrane Collaboration’s Tool for Assessing Risk of Bias in Randomized Trials [24]. Quality assessment was performed independently by two reviewers (HECS and CMS) and compared. Data were extracted from selected articles, and the answers were classified using the parameters “yes”, “no” or “unclear”. “Yes” corresponded to low risk of bias, “no” high risk of bias, and “unclear” unknown or unclear risk of bias.

According to the instrument used, assessments should consider the risk of material bias rather than any bias. “Material bias” was defined as bias of sufficient magnitude to have a notable impact on the results or conclusions of the trial, recognizing the subjectivity involved in judgment process [24]. In this case, articles with 75% of yes answers were considered low risk of bias, articles with 75% of “no” and/or “unclear” high risk of bias, and finally articles with 50% to 75% of “no” and/or “unclear” answers were considered of moderate risk of bias.

### Considered outcomes

The intervention effects were compared to the following outcomes: inflammatory markers, preterm birth (< 37 weeks), low birth weight (< 2.500 g), and preeclampsia (140–160 to 90–110 mm/Hg). The reduction of inflammatory markers after nonsurgical periodontal markers was also correlated to APOs.

### Meta-analysis

For the meta-analysis, the risk ratios of incidences were calculated for each one of the available outcomes (preterm birth < 37 weeks, low birth weight, preeclampsia), at 95% significance level. Heterogeneity and weight of the studies were calculated. Meta-analysis was performed by the 5.3.5 version of Review Manager software (Nordic Cochrane Center, Copenhagen, Denmark).

## Results

### Search findings

**Fig. 1:**
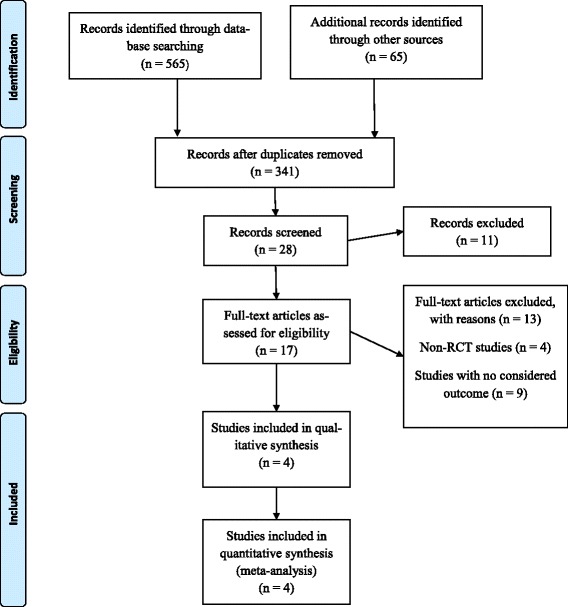
Flow diagram of literature search and selection criteria

The electronic search of five databases resulted in 565 references. Removal of duplicated studies resulted in 326 references. Titles and abstracts from these studies were read and those not fulfilling the eligibility criteria were excluded. In addition, gray literature was searched. ProQuest returned 5 references and Open Grey none. From Google Scholar, the first 60 references were considered for evaluation. At the end of phase 1, 28 studies remained for full text reading (phase 2). Manual search of reference lists did not provide additional studies. Full text reading resulted in 4 eligible studies for qualitative analysis. Appendix II presents excluded articles and reasons for exclusion (see Additional file 3). A flowchart of the complete process is shown in Fig. 1.

Included studies were conducted in Ireland [13], Australia [16], USA [22], and India [23]. All included articles were published in English.

All four selected articles were randomized clinical trials that evaluated several periodontal inflammatory biomarkers from different sources: IL-1β, IL-6, IL-8, IL-10, IL-12p70, IL-17A, MCP-1, and TNF-α, PGE2, d-8-iso, PGF2a, IL-1β, and IL-6 from GCF [16, 19]; C-reactive protein (CRP) [22, 23], sICAM-1, sGP-130, IL-6 sr, d-8-iso, and PGF2a [22] from blood serum; and IL-1β, IL-6, and IL-8 from umbilical cord serum [13].

All papers evaluated PTB [13, 16, 22, 23], and two papers assessed LBW [13, 23] as APO. None of them considered preeclampsia as gestational outcome. Table 1 summarizes details of studies regarding population, interventions, outcomes, and conclusions.

**Table 1:** Summary of descriptive characteristics of included articles (*n* = 4)

Author, year, country	Age in yearsmean ± SD and/or range	Case definitions (PD and PTB/LBW)	Groups (n*) and treatments	Source biomarkers	Outcomeincidence of PTB/ LBW/preeclampsia *n* (%) and *p* value inflammatory markers between groups—*p* value	Main conclusions	Risk of bias assessments
Offenbacher et al., 2006, USA	> 18 years old treated 26.8 ± 5.5. untreated 25.7 ± 5.4 *p* > 0.05.	PD: two or more sites measuring ≥ 5-mm probing depths plus periodontal attachment loss of 1 to 2 mm at one or more sites with PDs ≥ 5 mm.PTB: delivery < 37 weeks.	Treated (*n* = 40) periodontal scaling and root planing and polishing. Oral health instructions. Untreated (*n* = 34) supragingival debridement. Periodontal treatment and oral health instructions after delivery.	GCF PGE2, d-8-iso PGF_2a_, IL-1β, and IL-6 at first and last dental examinations. Serum sICAM-1, sGP-130, IL-6 sr, d-8-iso, PGF_2a_, and CRP at first obstetric visit and at delivery.	PT:Treated: 9/35 (25, 7%)Untreated: 14/32 (43.8%) *p* = 0.026 Preeclampsia^p^ Treated: 1/40 (2.5%) Untreated: 2/34 (5.9%) GCF IL-1β—*p* = 0.01 (lower on treated group) PGE2, [iso] PGE_2a_, and IL-6—*p* > 0.05 Serum IL-6 sr—*p* = 0.03 (lower on treated group) IL-6, sICAM-1, d-8-iso PGE_2a_, soluble glycoprotein-130 (sGP-130)^n^, and CRP—*p* > 0.05.	This pilot study provides further evidence supporting the potential benefits of periodontal treatment on pregnancy outcomes. Treatment was safe, improved periodontal health, and prevented periodontal disease progression. Preliminary data show a 3.8-fold reduction in the rate of preterm delivery, a decrease in periodontal pathogen load, and a decrease in both GCF IL-1β and serum markers of IL-6 response.	Moderate
Pirie et al., 2013, Ireland	> 18 years old treated: 30.5 ± 4.5. untreated: 30.5 ± 5.5.	PD: PPD ≥ 4 mm at ≥ 4 sites and CAL ≥ 2 mm at ≥ 4 sites. PTB < 37 weeks. LBW < 2500 g.	Treated (*n* = 49) nonsurgical periodontal therapy. Untreated (*n* = 50) oral hygiene instruction and supragingival cleaning of all teeth at baseline.	Serum cord IL-1β, IL-6, and IL-8 at delivery.	PTB Treated: 4/49 (8.2%) Untreated: 1/50 (2%) *p* > 0.05 LBW Treated: 3/49 (6.2%) Untreated: 1/50 (2%) *p* > 0.05 (IL)-1β, IL-6 and IL-8 between groups *p* > 0.05.	Intra-pregnancy nonsurgical periodontal treatment, completed at 20 to 24 weeks, did not reduce the risk of preterm, low-birth-weight delivery in this population.	Low
Khairnar et al., 2015, India	17 to 35 years old (mean and SD not available).	PD: > 2 mm CAL: at > 50% examined sites. PTB: < 37 weeks. LBW: < 2500 g.	Treated (*n* = 50) scaling and root planing with 0.2% chlorhexidine rinse once a day. Untreated (*n* = 50) same treatment after delivery.	Serum CRP at baseline and after delivery.	PTB Treated: 16/50 (32%) Untreated: 36/50 (72%) *p* < 0.05 LBW Treated: 18/50 (36%) Untreated: 26/50 (52%) *p* < 0.05 CRP Treated: reduction (*p* < 0.05) Untreated: no reduction (*p* > 0.05).	Nonsurgical supportive periodontal therapy can significantly reduce the risk of PTB and LBW deliveries. Nonsurgical supportive periodontal therapy can reduce raised serum CRP levels in pregnant females affected with periodontitis.	High
Penova-Vaselinovic et al., 2015, Australia	> 16 years old treated 31.9 ± 5.4. untreated31.7 ± 5.0 *p* = 0.651.	PD: ≥ 3.5 mm PPD at 25% of sites. PTB: < 37 week.	Treated (*n* = 40) nonsurgical debridement of the sub- and supra-gingival plaque and removal of calculus and overhanging restoration adjustments. Untreated (*n* = 39) same treatment after delivery.	GCF IL-1β, IL-6, IL-8, IL-10, IL-12p70, IL-17A, MCP-1, and TNF-α at 20 and 28 weeks of gestation.	PTB Treated: 5/40 (12.5%) Untreated: 4/39 (10.3%) *p* = 0.754 GCF IL-1β, IL-10, IL-12p70, IL-17 and IL-6—*p* < 0.05 (lower on treated group) MCP-1, IL-8 and TNF-α: *p* < 0.05 (increased on treated group).	PD treatment in pregnancy reduces the levels of some inflammatory mediators in the GCF and improves dental parameters, with no overt effects on pregnancy outcome.	Moderate

*PD* periodontal disease, *PTB* preterm birth, *LBW* low birth weight, *GCF* gingival crevicular fluid, *CRP* C-reactive protein, *PPD* probing pocket depth, *CAL* clinical attachment level, *PGR2* prostaglandin E2, *IL* interleukine, *TNF* tumor necrosis factor, *MCP-1* monocyte chemoattractant protein 1, *sICAM-1* soluble intracellular adhesion molecule 1, *sGP-130* soluble glycoprotein-130, *d-8-iso* d-8-isoprostane [iso], n* considered at last clinical examination, after computed dropouts

### Risk of bias within studies

None of the studies fulfilled all methodological quality criteria. One study [13] was considered at low risk of bias, while one [23] was considered at high risk of bias. The other two [16, 22] were considered at moderated risk of bias.

Three studies [16, 22, 23] did not describe the randomization process properly, nor the allocation concealment.

No study described blinding of subjects, researchers and outcome evaluators, as determined by the risk of bias instrument used, and even when only blinding of outcome evaluators was considered, one single study [22] mentioned it, with no further description.

One study [23] did not clearly report the pre-determined main outcome and was not considered free of other problems that could put it at high risk of bias.

**Fig. 2:**
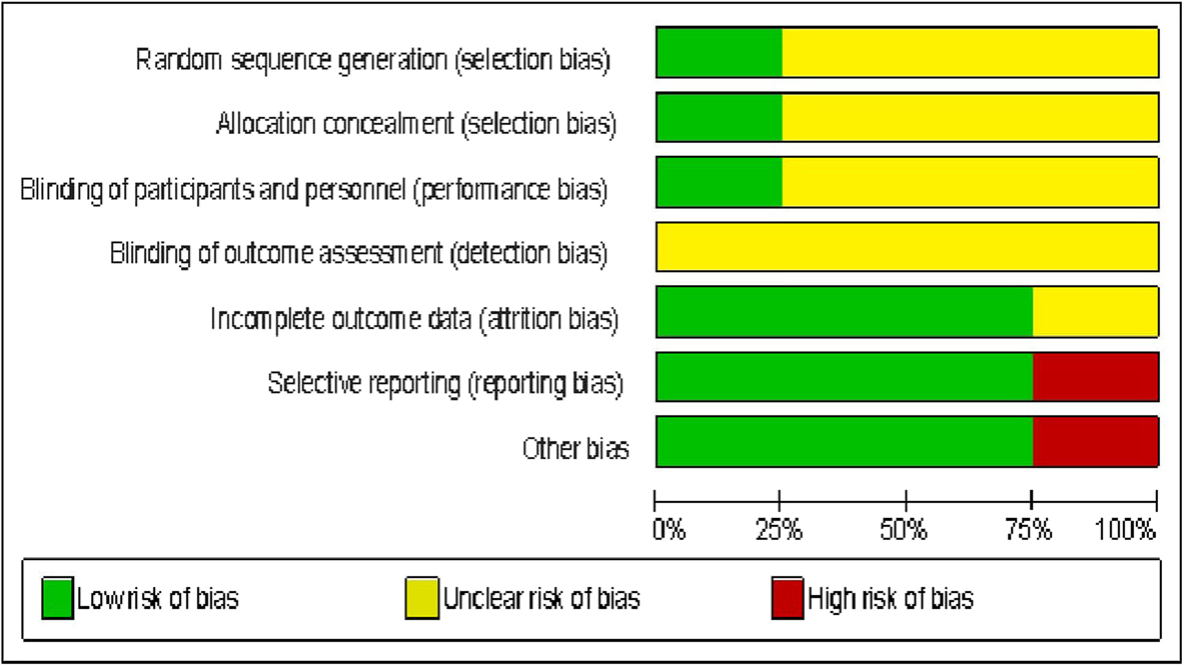
Risk of bias graph, authors’ judgements about each risk of bias item presented as percentages across all four included studies

More information about the risk of bias can be found in Fig. 2 (summarized assessment) and Appendix III (see Additional file 4) [24].

### Results of individual studies

Offenbacher et al. [22] reported pregnant women with periodontitis who received nonsurgical periodontal therapy showed significant lower GCF levels of IL-1β (*p* = 0.01) and serum levels of IL-6 sr (*p* = 0.03) in postpartum. Within untreated pregnant women, many mediators showed increased serum levels during gestation, including IL-6, sICAM1, d-8-iso, sGP130, IL-6 sr, and CRP. There was less PTB occurrence among treated women (*p* = 0.026).

Pirie et al. [13] did not find significant differences between treated and untreated groups for IL-8, IL-1β, and IL-6 from cord blood, although all clinical parameters recorded, periodontal pocket depth (PPD), clinical attachment level (CAL), and bleeding on probing (BOP), showed statistically significant improvement after nonsurgical periodontal therapy. IPPT increased the occurrence of PTB and LBW (statistically nonsignificant).

Khairnar et al. [23] showed significant reduction of CRP levels in blood serum from treated group (*p* < 0.05), compared to the baseline value, while the same was not observed for untreated group. IPPT reduced PTB and LBW (*p* < 0.05).

Penova-Veselinovic et al. [16] observed that treated group showed significantly lower levels of IL-1β, IL-10, IL-12p70, and IL-6 in GCF compared with untreated group. However, levels of MCP-1 and TNF-α increased only in the treated group. The reduction in GCF cytokine levels was also significant when the values before and after periodontal treatment within the treated group were compared. There was no difference between PTB among treated and untreated women.

### Synthesis of results

The four included studies contemplated 349 patients (174 in the treated group and 175 in the untreated group). All studies reported preterm birth as a primary outcome. From 174 pregnant women with periodontal disease who underwent nonsurgical periodontal therapy, 30 (17.2%) delivered preterm babies, and from 175 pregnant women with untreated periodontal disease, 54 (30.8%) delivered preterm babies.

Two studies [13, 23] reported LBW as a primary outcome. These studies assessed 199 births, of which 18 (9.04%) in treated group and 27 (13.5%) in untreated group resulted in LBW babies.

None of the studies evaluated preeclampsia as the primary outcome. Two studies showed relation between serum biomarker level reduction after IPPT and lower rates of APO occurrence [22, 23], one for IL-6 sr [22] and other for CRP [23].

### Meta-analysis

Studies were grouped accordingly to primary outcome, preterm birth, or low birth weight, and meta-analysis was performed. Heterogeneity was calculated by inconsistency indexes (I2), and values were below 50%; therefore, fixed effect was considered for analysis.

**Fig. 3:**
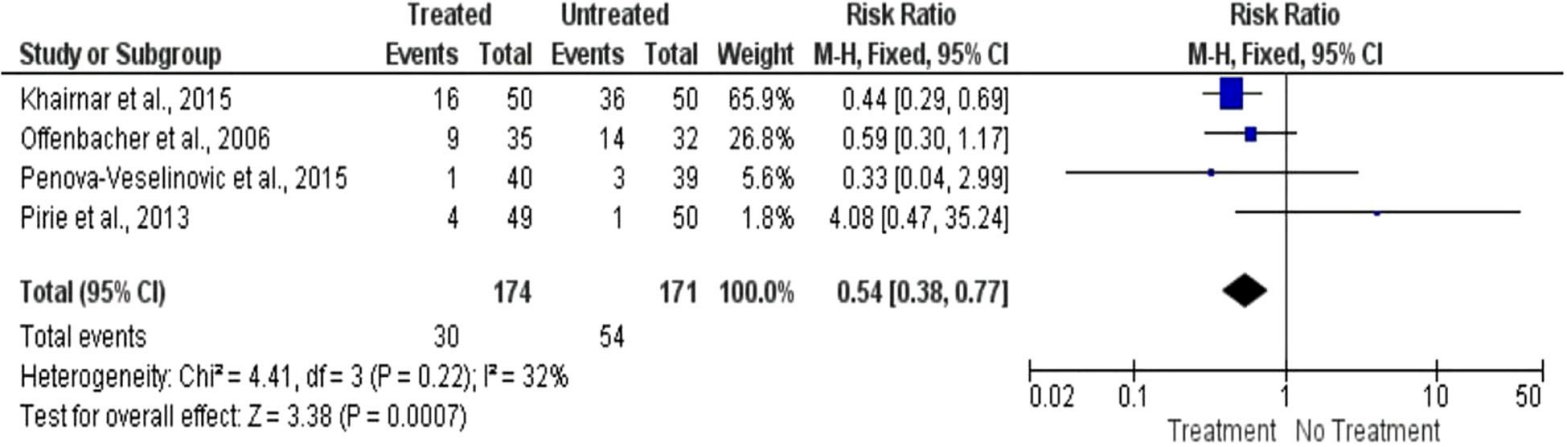
Forest plot for premature birth outcome between treated and untreated pregnant women

**Fig. 4:**
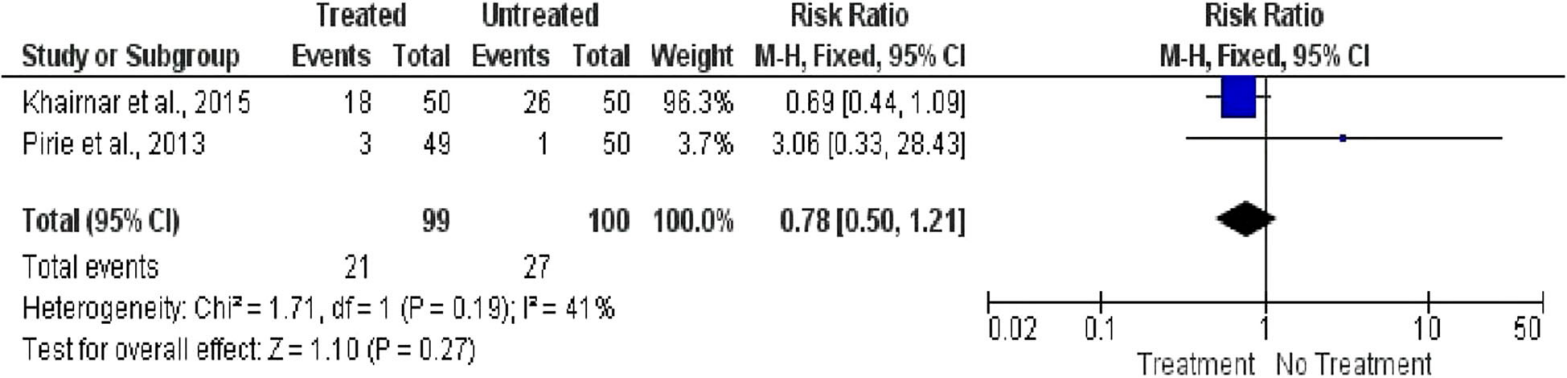
Forest plot for low birth weight outcome between treated and untreated pregnant women

Studies were clustered into preterm birth (Fig. 3) and low birth weight (Fig. 4). Additional information on this meta-analysis of risk ratio (RR) can be found on Figs. 3 and 4.

The meta-analysis of four studies [13, 16, 22, 23] showed a tendency for reduction of the risk of preterm birth before 37 weeks for treated group, RR = 0.54, 95%CI 0.38–0.77; *p* = 0.0007; I2 32% (Fig. 3).

The meta-analysis of two studies [13, 23] did not show relation between nonsurgical periodontal therapy during pregnancy and the low birth weight, RR = 0.78, 95% CI 0.50–1.21; *p* = 0.27; I2 41% (Fig. 4).

## Discussion

This systematic review and meta-analysis evaluated the effect of IPPT on periodontal inflammatory biomarkers and APO. Included studies showed IPPT reduced inflammatory biomarker level from GCF and some from blood serum and improved periodontal clinical parameters (PPD, CAL, and BOP). Two included studies [22, 23] showed a positive relation between reduced serum levels of inflammatory markers and lower premature birth (PB)/low birth weight (LBW) rates.

Intra-pregnancy nonsurgical periodontal therapy, once reducing periodontal pathogens and consequently the inflammatory response, could theoretically decrease APO risk. However, besides IPPT reduced inflammatory biomarkers from GCF [16, 22], it did not remarkably reduce inflammatory biomarker level from blood serum [22], except CRP [23], and did not influence biomarker level from cord blood [13]. Therefore, apparently it was ineffective in stopping inflammatory cascade triggered by periodontitis and intensified by pregnancy.

At the meta-analysis performed, one study [23] showed strong statistical significance for decrease of premature birth rate after nonsurgical periodontal therapy. Two studies [16, 22] showed no difference, and the last one [13] showed 4.08-fold increased risk for premature birth occurrence after intra-pregnancy periodontal therapy (RR = 4.08; 95% CI [0.47–35.24]).

During overall analysis of included studies, however, it was observed tendency for protection of premature births (PB) occurrence after intra-pregnancy nonsurgical periodontal therapy (RR = 0.54; 95% CI [0.38 to 0.77]; *p* = 0.0007). Besides, two studies [13, 23] showed highly antagonistic results for effect of nonsurgical periodontal therapy on PB. Khairnar et al. [23] had higher weight on analysis (65.9%), compared to Pirie et al. [13] (1.8%). However, methodological issues, as reduced sample size, confounding factors, and high risk of bias [23] affected study comparison and this relationship, so results must be considered cautiously.

Regarding low birth weight (LBW), two studies [13, 23] showed no statistical difference at meta-analysis (RR = 0.78; 95% CI [0.50–1.21]; *p* = 0.27). Khairnar et al. [23] had higher weight (96.3%) for protective effect of periodontal therapy on LBW occurrence, and Pirie et al. [13] showed 3.06-fold increased risk for LBW after intra-pregnancy nonsurgical periodontal therapy (RR = 3.06, 95% CI [0.33–28.43]). Once again, due to methodological issues and high risk of bias observed [23], results must be considered cautiously.

In this systematic review, two studies [22, 23] showed statistical significance for decrease of premature birth rate after IPPT. These papers also showed reduction for CRP [23] and IL-6 sr [22] from serum blood after IPPT. Pregnancy alters hormonal and immunological responses, leading to increased local periodontal inflammatory response for the same microbiological challenge, once the dental plaque increases in volume during pregnancy, without significant composition changes. Higher level of IL-6 at GCF during pregnancy is accompanied by systemic activation of IL-6. Even if serum increase of IL-6 is not significant, effects on pregnancy might be expressive, once the whole IL-6 cascade activation is observed (sICAM1, sgp130 e IL-6 sr) [22].

A cohort prospective study [25] showed periodontitis increases the level of CRP during pregnancy. Serum blood CRP could be one of the plausible inflammatory biomarkers in the association between periodontitis and APO. Periodontitis acts as a reservoir of bacterial lipopolysaccharides (LPS), which induces the production of CRP [26]. CRP is synthesized in the liver from hepatic action of inducers such as IL-6 and IL-8iso [26], and the maternal systemic stress during pregnancy leads to activation of hepatic route. Elevated CRP levels could increase the inflammatory response by activation of the complement system, release of inflammatory cytokines, resulting in premature rupture of the fetal-placental membrane [27, 28]. Thus, the hypothesis that nonsurgical periodontal therapy might reduce the bacterial load and associated bacteremia, therefore reducing the inflammatory stress during pregnancy and IL-6 cascade, contributes to reduction in CRP levels and, consequently, the incidence of APO [22, 23]. In this review, two studies tested CRP from serum, with opposite results. Khairnar et al. [23] observed reduction of CRP level after IPPT, and Offenbacher et al. [22] found no difference for CRP, but positive for IL-6rs. Both studies observed less PTB after IPPT.

Labor natural process involves liver production of inflammatory mediators during pregnancy that gradually rises till leads to rupture of the membranes, culminating with labor. However, host immune response to chronic periodontitis produces inflammatory mediators that theoretically could stimulate the hepatic route earlier, leading to increased levels of inflammatory mediators, accelerating labor, and causing premature delivery or low birth weight [15]. So, the control of inflammatory biomarker level after IPPT would be an easy way to attest success of therapy and monitor labor evolution.

However, there are conflicting results about correlation between local (GCF/saliva) and systemic levels of inflammatory periodontal biomarkers in women with chronic periodontitis. High cytokine levels were found in GCF of pregnant women with widespread periodontal inflammation but limited periodontal destruction, still, local cytokine levels were not strongly associated with serum levels [29]. Nevertheless, a recent study with a bigger sample of preconception women with periodontitis showed a positive association between elevated concentrations of IL-1β, β-glucuronidase, and TNF-α in saliva and serum [18]. And a study with post-delivery women with periodontitis found an association between serum and GCF levels of TNF-α and PGE2 [19].

Conflicting results were also observed regarding effects of nonsurgical periodontal therapy on systemic inflammatory biomarkers, besides the reduction of local levels on GCF [16, 17, 30]. Some studies found no significant effect on serum inflammatory biomarkers [10, 17], while others found significant reduction [22, 23]. In opposite, increased serum levels of MCP-1, TNF-α, and IL-8 after IPPT were found in one study [16]. This increase may be attributed to differences in individual responses to inflammatory periodontal disease and its treatment [31], or may reflect an increase related to gestational age [16].

The lack of consistent results for IPPT on systemic inflammatory biomarker reduction may be a consequence of the moment of periodontal treatment. It is possible that to diagnose and subsequently treat maternal periodontitis during pregnancy is already too late to halt inflammatory cascade and improve pregnancy outcomes [32]. In fact, all included studies treated pregnant women between 11 and 24 gestational weeks, because, during pregnancy, second trimester is the safest period to periodontal treatment. There is no publicized study about effects of nonsurgical periodontal therapy prior to conception on APO occurrence, although a research on this subject is currently on course [18].

Another possible explanation for conflictive results of IPPT on periodontal inflammatory biomarkers and APO among studies is sample characteristics. Studies in which treatment during pregnancy were effective were conducted among Indian [23] or African-American [22] populations at high risk of preterm delivery, as described before in previous studies [33–35], and therefore not generalizable to Caucasian populations, as pointed by Pirie et al. [13]. Samples studied by Khairnar et al. [23] and Offenbacher et al. [22] were nonwhite, from mixed ethnicity, with low socioeconomic status, and both studies found reduction of PTB after IPPT. The other two included studies, one [16] used population with low socioeconomic status from mixed ethnicity, the other [13] was conducted with a less mixed population although unbalanced for socioeconomic status, and both found no positive effect of IPPT on APO occurrence. Apparently, some populations/ethnicities should be more prone to benefit from IPPT to reduce occurrence of APO.

An important limitation among the selected papers that should be analyzed is the definition of chronic periodontitis. Chronic periodontitis is an infectious disease resulting in inflammation within the supporting tissues of the teeth, progressive attachment, and bone loss and is characterized by pocket formation and/or gingival recession. It is recognized as the most frequently occurring form of periodontitis. It is prevalent in adults but can occur at any age. The disease is usually associated with the presence of plaque and calculus. Progression of attachment loss usually occurs slowly, but periods of rapid progression can occur [36]. Although this is the American Academy of Periodontology current definition, there is no certainty about included study concepts about chronic periodontitis. For inclusion criteria, author statement at methodology was accepted, but it is not impossible that other forms of periodontitis could be included at studies samples.

Another point of concern is periodontitis case-definition. Currently, there is no single and universally accepted case-definition for chronic periodontitis. Two current case definitions are widely used, one European and another American. The case-definition proposed by Tonetti & Claffey [37] in Group C of the European Workshop in Periodontology mentions a sensitive definition as “presence of proximal attachment loss ≥3 mm in two or more nonadjacent teeth” and an extensive definition with “presence of proximal attachment loss ≥5 mm in ≥30% teeth”. The American definition proposed by Page & Eke [38] from the criteria of the Center for Disease Control/American Academy of Periodontology defines mild periodontitis as “at least two interproximal sites with clinical attachment level (CAL) ≥3mm and at least two interproximal sites with probing depth (PD) ≥4mm (not on the same tooth) OR one site with 5mm PD”; moderate periodontitis as “at least two interproximal sites with CAL ≥4mm (not on the same tooth) OR at least two interproximal sites with PD ≥5mm (not on the same tooth)”; and severe periodontitis as “at least two interproximal sites with CAL ≥6mm (not on the same tooth) OR at least one interproximal site with PD ≥5mm”. None of included studies used any of these definitions. Some used more conservative definitions [16, 23], compatible with mild periodontitis, while other used more extensive definitions [13, 22], compatible with moderate periodontitis. This finding may contribute to confound results.

Beyond the case definition of chronic periodontitis, some limitations of this study include scarce data for LBW as primary outcome, absence of large RCT testing inflammatory biomarkers before and after IPPT and correlating then with APOs occurrence, and great heterogeneity of inflammatory biomarkers tested in available studies, as well as diversity of source (serum blood, cord blood, and GCF). Due to these limitations, further studies are necessary to confirm association between biomarker level after IPPT and APO occurrence rate.

## Conclusion

Intra-pregnancy nonsurgical periodontal therapy improved periodontal clinical parameters, decreased periodontal inflammatory biomarker levels from GCF and some from serum blood; however, it did not influence inflammatory biomarker level from cord blood nor consistently reduced preterm birth or low birth weight occurrence.

## Additional files

Additional file 1: Prisma Checklist. PRISMA Checklist used by the authors to assess the selected systematic reviews. The 27 checklist items pertain to the content of a systematic review and meta-analysis, which include the title, abstract, methods, results, discussion, and funding. (DOC 64 kb)

Additional file 2: Appendix I presents the search strategy used. Database search strategy. Electronic search performed in the databases used in the research. (DOC 56 kb)

Additional file 3: Appendix II presents the excluded articles and reasons for exclusion. Excluded articles and reasons for exclusion (*n* = 24). List of articles that did not fulfill the eligibility criteria in phase 2. (DOC 46 kb)

Additional file 4: Appendix III presents the risk of bias in select papers.Risk of bias assessed by The Cochrane Collaboration’s tool for assessing risk of bias. Assessment of bias risk in the selected studies in the search strategy. (DOC 33 kb)

## Abbreviations

APO: Adverse pregnancy outcomes
BOP: Bleeding on probing
CAL: Clinical attachment level
CRP: C-reactive protein
d-8-iso: d-8-Isoprostane [iso]
GCF: Gingival crevicular fluid
IL: Interleukine
IPPT: Intra-pregnancy nonsurgical periodontal therapy
LBW: Low birth weight
LILACS: Latin American and Caribbean Literature
LPS: Lipopolysaccharides
MCP-1: Monocyte chemoattractant protein 1
MeSH: Medical Subject Headings
PD: Periodontal disease
PGE2: Prostaglandin E2
PICO: Participants, intervention, comparator, outcome
PPD: Periodontal pocket depth
PRISMA: Preferred Reporting Items for Systematic Reviews and Meta-Analyses
PROSPERO: International prospective register of systematic reviews
PTB: Premature birth
PUBMED: International Literature in Health Science
RCT: Randomized controlled trial
sGP-130: Soluble glycoprotein-130
sICAM-1: Soluble intracellular adhesion molecule 1
TNF-α: Tumor necrosis factor alpha
