# Supplementary material for: Effect of intra-pregnancy nonsurgical periodontal therapy on inflammatory biomarkers and adverse pregnancy outcomes: a systematic review with meta-analysis
Source: Syst Rev. 2017 Oct 10;6:197. doi: 10.1186/s13643-017-0587-3 (PMC5635531; doi:10.1186/s13643-017-0587-3)
Supplement: Supplementary file 2 — Appendix I presents the search strategy used. Database search strategy. Electronic search performed in the databases used in the research. (DOC 56 kb) [file 13643_2017_587_MOESM2_ESM.doc]

**Appendix 1.** Database search strategy.

| **Database** | **Search ( june 5^th^ 2017)** | **References** |
| --- | --- | --- |
| **PubMed** | **#1 -** ("pregnant women"[MeSH Terms] OR "pregnant women"[All Fields] OR "pregnant woman"[All Fields] OR ("pregnancy"[MeSH Terms] OR "pregnancy"[All Fields]) OR ("pregnancy"[MeSH Terms] OR "pregnancy"[All Fields] OR "pregnancies"[All Fields]) OR ("gravidity"[MeSH Terms] OR "gravidity"[All Fields]) OR ("gravidity"[MeSH Terms] OR "gravidity"[All Fields] OR "gravidities"[All Fields]) OR ("gravidity"[MeSH Terms] OR "gravidity"[All Fields] OR "pregnant"[All Fields]) OR pregnants[All Fields] OR ("pregnancy"[MeSH Terms] OR "pregnancy"[All Fields] OR "gestation"[All Fields]) OR ("female"[MeSH Terms] OR "female"[All Fields] OR "females"[All Fields]))  **#2** - ("chronic periodontitis"[MeSH Terms] OR "chronic periodontitis"[All Fields] OR "adult periodontitis"[All Fields] OR ("periodontitis"[MeSH Terms] OR "periodontitis"[All Fields]) OR "periodontal disease"[All Fields] OR "periodontal therapy"[All Fields] OR "periodontal treatment"[All Fields] OR "scaling and root planing"[All Fields] OR "periodontal intervention"[All Fields] OR "dental scaling"[All Fields] OR "root planing"[All Fields] OR "subgingival curettage"[All Fields] OR "root scaling"[All Fields] OR "subgingival scaling"[All Fields] OR "supragingival Scaling"[All Fields] OR "periodontal therapeutics"[All Fields] OR (periodontal[All Fields] AND ("therapeutics"[MeSH Terms] OR "therapeutics"[All Fields] OR "attachment loss, periodontal"[All Fields] OR "loss, periodontal attachment"[All Fields] OR "periodontal debridement"[All Fields] OR ("periodontal debridement"[MeSH Terms] OR ("periodontal"[All Fields] AND "debridement"[All Fields])) OR "nonsurgical periodontal debridement"[All Fields] OR ("nonsurgical"[All Fields] AND "periodontal"[All Fields] AND "debridement"[All Fields])) OR "nonsurgical periodontal therapy"[All Fields]))  **#3 -** ("inflammatory markers"[All Fields] OR "inflammatory mediators"[All Fields] OR "serum markers"[All Fields] OR "cytokine"[All Fields] OR "cytokines"[MeSH Terms] OR "cytokines"[All Fields] OR "crevicular fluid, gingival"[All Fields] OR ("gingival crevicular fluid"[MeSH Terms] OR ("gingival"[All Fields] AND "crevicular"[All Fields] AND "fluid"[All Fields]) OR "gingival crevicular fluid"[All Fields] OR ("crevicular"[All Fields] AND "fluids"[All Fields] AND "gingival"[All Fields])) OR ("gingival crevicular fluid"[MeSH Terms] OR ("gingival"[All Fields] AND "crevicular"[All Fields] AND "fluid"[All Fields]) OR "gingival crevicular fluid"[All Fields] OR ("fluid"[All Fields] AND "gingival"[All Fields] AND "crevicular"[All Fields])) OR ("gingival crevicular fluid"[MeSH Terms] OR ("gingival"[All Fields] AND "crevicular"[All Fields] AND "fluid"[All Fields]) OR "gingival crevicular fluid"[All Fields] OR ("fluids"[All Fields] AND "gingival"[All Fields] AND "crevicular"[All Fields])) OR "gingival crevicular fluids"[All Fields] OR "interleukin 1beta"[All Fields] OR "interleukin-1 beta"[All Fields] OR "interleukin 1 beta"[All Fields] OR "IL-1 beta"[All Fields] OR "tumor necrosis factor alpha"[All Fields] OR "TNFalpha"[All Fields] OR "TNF-alpha"[All Fields] OR "tumor necrosis factor"[All Fields] OR "markers, biological"[All Fields] OR "biomarkers"[All Fields] OR "marker biological"[All Fields] OR "biological marker"[All Fields] OR "biologic marker"[All Fields] OR "biomarkers"[MeSH Terms] OR "biological markers"[All Fields] OR ("marker"[All Fields] AND "biologic"[All Fields])) OR "biologic markers"[All Fields] OR ("markers"[All Fields] AND "biologic"[All Fields]) OR "C reactive protein"[All Fields] OR "protein, C-reactive"[All Fields] OR "mediators, inflammation"[All Fields] OR "mediators of inflammation"[All Fields])  **#4** - (premature[All Fields] OR "premature birth"[MeSH Terms] OR "premature birth"[All Fields] OR preterm[All Fields] OR "preterm birth"[All Fields] OR "obstetric labor, premature"[MeSH Terms] OR "infant, premature"[MeSH Terms] OR ("infant, premature"[MeSH Terms] OR ("infant"[All Fields] AND "premature"[All Fields]) OR "premature infant"[All Fields] OR "prematurity"[All Fields]) OR "infant, low birth weight"[MeSH Terms] OR "low birth weight infant"[All Fields] OR "low birth weight"[All Fields] OR "preterm delivery"[All Fields] OR ("pre-eclampsia"[MeSH Terms] OR "pre-eclampsia"[All Fields] OR ("pre"[All Fields] AND "eclampsia"[All Fields]) OR "pre eclampsia"[All Fields]) OR "pre eclampsia"[All Fields] OR ("pre-eclampsia"[MeSH Terms] OR "pre-eclampsia"[All Fields] OR "preeclampsia"[All Fields]) OR "pre-eclampsia"[MeSH Terms] OR "pregnancy toxemias"[All Fields] OR "pregnancy toxemia"[All Fields] OR "complication, pregnancy"[All Fields] OR "pregnancy complication"[All Fields] OR "complications, pregnancy"[All Fields] OR "outcomes, pregnancy"[All Fields] OR "pregnancy outcomes"[All Fields] OR ("pregnancy outcome"[MeSH Terms] OR ("pregnancy"[All Fields] AND "outcome"[All Fields]) OR "pregnancy outcome"[All Fields] OR ("outcome"[All Fields] AND "pregnancy"[All Fields])))  **#5 -** (#1 AND #2 AND #3 AND #4) | **147** |
| **Cochrane** | "pregnant women" OR "pregnant woman" OR pregnancy OR pregnancies OR gravidity OR gravidities OR pregnant OR pregnants OR gestation OR females in Title, Abstract, Keywords and "chronic periodontitis" OR "adult periodontitis" OR periodontitis OR "periodontal disease" OR "periodontal therapy" OR "periodontal treatment" OR "scaling and root planing" OR "periodontal intervention" OR "dental scaling" OR "root planing" OR "subgingival curettage" OR "root scaling" OR "subgingival scaling" OR "supragingival scaling" OR "periodontal therapeutics" OR "loss, periodontal attachment" OR "attachment loss, periodontal" OR "periodontal debridement" OR "nonsurgical periodontal debridement" OR "nonsurgical periodontal therapy" in Title, Abstract, Keywords and "inflammatory markers" OR "inflammatory mediators" OR "serum markers" OR cytokines OR cytokine OR "crevicular fluid, gingival" OR "crevicular fluids, gingival" OR "fluid, gingival crevicular" OR "fluids, gingival crevicular" OR "gingival crevicular fluids" OR "interleukin 1beta" OR "interleukin-1 beta" OR "interleukin 1 beta" OR "IL-1 beta" OR "tumor necrosis factor alpha" OR "TNFalpha" OR "TNF-alpha" OR "tumor necrosis factor" OR "markers, biological" OR "biomarkers" OR "marker biological "OR "biological marker" OR "biologic marker" OR "marker, biologic" OR "biological markers" OR "biologic markers" OR "markers, biologic" OR "C reactive protein" OR "protein, C-reactive" OR "mediators, inflammation" OR "mediators of inflammation" in Title, Abstract, Keywords and premature OR "premature birth" OR preterm OR "preterm birth" OR "obstetric labor, premature" OR "infant, premature" OR prematurity OR "infant, low birth weight" OR "low birth weight infant" OR "low birth weight" OR "preterm delivery" OR pre-eclampsia OR "pre eclampsia" OR preeclampsia OR "pre-eclampsia" OR "pregnancy toxemias" OR "pregnancy toxemia" OR "complication, pregnancy" OR "Pregnancy complication" OR "complications, pregnancy" OR "outcomes, pregnancy" OR "pregnancy outcomes" OR "outcome, pregnancy" in Trials' | **9** |
| **Scopus** | ( TITLE-ABS-KEY ("pregnant women" OR "pregnant woman" OR pregnancy OR pregnancies OR gravidity OR gravidities OR pregnant OR pregnants OR gestation OR females) ) AND ( TITLE-ABS-KEY ("chronic periodontitis" OR "adult periodontitis" OR periodontitis OR "periodontal disease" OR "periodontal therapy" OR "periodontal treatment" OR "scaling and root planing" OR "periodontal intervention" OR "dental scaling" OR "root planing" OR "subgingival curettage" OR "root scaling" OR "subgingival scaling" OR "supragingival scaling" OR "periodontal therapeutics" OR "periodontal attachment loss" OR "periodontal debridement" OR "nonsurgical periodontal debridement" OR "nonsurgical periodontal therapy") ) AND ( TITLE-ABS-KEY ("inflammatory markers" OR "inflammatory mediators" OR "serum markers" OR cytokines OR cytokine OR "crevicular fluid, gingival" OR "crevicular fluids, gingival" OR "fluid, gingival crevicular" OR "fluids, gingival crevicular" OR "gingival crevicular fluids" OR "interleukin 1beta" OR "interleukin-1 beta" OR "interleukin 1 beta" OR "IL-1 beta" OR "tumor necrosis factor alpha" OR "TNFalpha" OR "TNF-alpha" OR "tumor necrosis factor" OR "markers, biological" OR "biomarkers" OR "marker biological "OR "biological marker" OR "biologic marker" OR "marker, biologic" OR "biological markers" OR "biologic markers" OR "markers, biologic" OR "C reactive protein" OR "protein, C-reactive" OR "mediators, inflammation" OR "mediators of inflammation") ) AND ( TITLE-ABS-KEY (premature OR "premature birth" OR preterm OR "preterm birth" OR "obstetric labor, premature" OR "infant, premature" OR prematurity OR "infant, low birth weight" OR "low birth weight infant" OR "low birth weight" OR "preterm delivery" OR pre-eclampsia OR "pre eclampsia" OR preeclampsia OR "pre-eclampsia" OR "pregnancy toxemias" OR "pregnancy toxemia" OR "complication, pregnancy" OR "Pregnancy complication" OR "complications, pregnancy" OR "outcomes, pregnancy" OR "pregnancy outcomes" OR "outcome, pregnancy")) | **201** |
| **Web of Science** | Tópico: ("pregnant women" OR "pregnant woman" OR pregnancy OR pregnancies OR gravidity OR gravidities OR pregnant OR pregnants OR gestation OR females) AND Tópico: ("chronic periodontitis" OR "adult periodontitis" OR periodontitis OR "periodontal disease" OR "periodontal therapy" OR "periodontal treatment" OR "scaling and root planing" OR "periodontal intervention" OR "dental scaling" OR "root planing" OR "subgingival curettage" OR "root scaling" OR "subgingival scaling" OR "supragingival scaling" OR "periodontal therapeutics" OR "periodontal attachment loss" OR "periodontal debridement" OR "nonsurgical periodontal debridement" OR "nonsurgical periodontal therapy") AND Tópico: ("inflammatory markers" OR "inflammatory mediators" OR "serum markers" OR cytokines OR cytokine OR "crevicular fluid, gingival" OR "crevicular fluids, gingival" OR "fluid, gingival crevicular" OR "fluids, gingival crevicular" OR "gingival crevicular fluids" OR "interleukin 1beta" OR "interleukin-1 beta" OR "interleukin 1 beta" OR "IL-1 beta" OR "tumor necrosis factor alpha" OR "TNFalpha" OR "TNF-alpha" OR "tumor necrosis factor" OR "markers, biological" OR "biomarkers" OR "marker biological "OR "biological marker" OR "biologic marker" OR "marker, biologic" OR "biological markers" OR "biologic markers" OR "markers, biologic" OR "C reactive protein" OR "protein, C-reactive" OR "mediators, inflammation" OR "mediators of inflammation") AND Tópico: (premature OR "premature birth" OR preterm OR "preterm birth" OR "obstetric labor, premature" OR "infant, premature" OR prematurity OR "infant, low birth weight" OR "low birth weight infant" OR "low birth weight" OR "preterm delivery" OR pre-eclampsia OR "pre eclampsia" OR preeclampsia OR "pre-eclampsia" OR "pregnancy toxemias" OR "pregnancy toxemia" OR "complication, pregnancy" OR "Pregnancy complication" OR "complications, pregnancy" OR "outcomes, pregnancy" OR "pregnancy outcomes" OR "outcome, pregnancy") | **121** |
| **Lilacs (Portuguese and Spanish)** | (tw:("mulheres grávidas" OR "mulheres/gestantes" OR gestação OR gravidez OR gestante OR gestantes OR "mujeres embarazadas" OR embarazo)) AND (tw:("raspagem radicular" OR "raspagem subgengival" OR "periodontite crônica" OR periodontite OR "doenças periodontais" OR "raspagem corono-radicular" OR "raspagem/alisamento" OR "raspagem dentária" OR "terapia periodontal não cirúrgica" OR " terapia periodontal no quirurgica" OR "raspado dental" OR "raspado subgingival" OR "periodontitis crónica" OR periodontitis OR "enfermedades periodontales")) AND (tw:("parto prematuro" OR "trabalho de parto prematuro" OR "parto pré-termo" OR prematuro OR "baixo peso ao nascer" OR "recém-nascido de baixo peso" OR "pré-eclâmpsia" OR "toxemia" OR "mediadores da inflamação" OR "resultado da gravidez" OR "fator de necrose tumoral alfa" OR interleucina OR "trabajo de parto prematuro" OR "trabajo de parto pre término" OR prematuro OR "recién nacido de bajo peso" OR "preeclampsia" OR "toxemia" OR "mediadores de inflamación" OR "resultado del embarazo" OR "factor de necrosis tumoral alfa" OR interleucina)) AND (instance:"regional") AND ( db:("LILACS")) | **87** |
| **ProQuest** | all("pregnant women" OR "pregnant woman" OR pregnancy OR pregnancies OR gravidity OR gravidities OR pregnant OR pregnant OR gestation OR females) AND all("chronic periodontitis" OR "adult periodontitis" OR periodontitis OR "periodontal disease" OR "periodontal therapy" OR "periodontal treatment" OR "scaling and root planing" OR "periodontal intervention" OR "dental scaling" OR "root planing" OR "subgingival curettage" OR "root scaling" OR "subgingival scaling" OR "supragingival scaling" OR "periodontal therapeutics" OR "periodontal attachment loss" OR "periodontal debridement" OR "nonsurgical periodontal debridement" OR "nonsurgical periodontal therapy") AND all("inflammatory markers" OR "inflammatory mediators" OR "serum markers" OR cytokines OR cytokines OR "crevicular fluid, gingival" OR "crevicular fluids, gingival" OR "fluid, gingival crevicular" OR "fluids, gingival crevicular" OR "gingival crevicular fluids" OR "interleukin 1beta" OR "interleukin-1 beta" OR "interleukin 1 beta" OR "IL-1 beta" OR "tumor necrosis factor alpha" OR "TNFalpha" OR "TNF-alpha" OR "tumor necrosis factor" OR "markers, biological" OR "biomarkers" OR "marker biological " OR "biological marker" OR "biologic marker" OR "marker, biologic" OR "biological markers" OR "biologic markers" OR "markers, biologic" OR "C reactive protein" OR "protein, C-reactive" OR "mediators, inflammation" OR "mediators of inflammation") AND all(premature OR "premature birth" OR preterm OR "preterm birth" OR "obstetric labor, premature" OR "infant, premature" OR prematurity OR "infant, low birth weight" OR "low birth weight infant" OR "low birth weight" OR "preterm delivery" OR pre-eclampsia OR "pre eclampsia" OR preeclampsia OR "pre-eclampsia" OR "pregnancy toxemias" OR "pregnancy toxemia" OR "complication, pregnancy" OR "Pregnancy complication" OR "complications, pregnancy" OR "outcomes, pregnancy" OR "pregnancy outcomes" OR "outcome, pregnancy") | **5** |
| **Open Grey** | ("pregnant women" OR "pregnant woman" OR pregnancy OR pregnancies OR gravidity OR gravidities OR pregnant OR pregnants OR gestation OR females) AND ("chronic periodontitis" OR "adult periodontitis" OR periodontitis OR "periodontal disease" OR "periodontal therapy" OR "periodontal treatment" OR "scaling and root planing" OR "periodontal intervention" OR "dental scaling" OR "root planing" OR "subgingival curettage" OR "root scaling" OR "subgingival scaling" OR "supragingival Scaling" OR "periodontal therapeutics" OR "periodontal attachment loss" OR "periodontal debridement" OR "nonsurgical periodontal debridement" OR "nonsurgical periodontal therapy") AND ("inflammatory markers" OR "inflammatory mediators" OR "serum markers" OR cytokine OR cytokines OR "crevicular fluid, gingival" OR "crevicular fluids, gingival" OR "fluid, gingival crevicular" OR "fluids, gingival crevicular" OR "gingival crevicular fluids" OR "interleukin 1beta" OR "interleukin-1 beta" OR "interleukin 1 beta" OR "IL-1 beta" OR "tumor necrosis factor alpha" OR "TNFalpha" OR "TNF-alpha" OR "tumor necrosis factor" OR "markers, biological" OR "biomarkers" OR "marker biological" OR "biological marker" OR "biologic marker" OR "marker, biologic" OR "biological markers" OR "biologic markers" OR "markers, biologic" OR "C reactive protein" OR "protein, C-reactive" OR "mediators, inflammation" OR "mediators of inflammation") AND (premature OR "premature birth" OR "premature birth" OR preterm OR "preterm birth" OR "obstetric labor, premature" OR "infant, premature" OR prematurity OR "infant, low birth weight" OR "low birth weight infant" OR "low birth weight" OR "preterm delivery" OR pre-eclampsia OR "pre eclampsia" OR preeclampsia OR "pre-eclampsia" OR "pregnancy toxemias" OR "pregnancy toxemia" OR "complication, pregnancy" OR "pregnancy complication" OR "complications, pregnancy" OR "outcomes, pregnancy" OR "pregnancy outcomes" OR "outcome, pregnancy") | **0** |
| **Google Scholar** | With all words ("chronic periodontitis" OR "periodontal disease" OR "nonsurgical periodontal therapy" OR "periodontal treatment") AND ("inflammatory markers" OR "serum markers" OR "cytokine" OR" "IL-1 beta" OR "TNF-alpha") AND ("preterm birth" OR "low birth weight" OR Pre-Eclampsia)  With the exact term ("pregnant woman") | **60** |
