# Supplementary material for: Effect of intra-pregnancy nonsurgical periodontal therapy on inflammatory biomarkers and adverse pregnancy outcomes: a systematic review with meta-analysis
Source: Syst Rev. 2017 Oct 10;6:197. doi: 10.1186/s13643-017-0587-3 (PMC5635531; doi:10.1186/s13643-017-0587-3)
Supplement: Supplementary file 3 — Appendix II presents the excluded articles and reasons for exclusion. Excluded articles and reasons for exclusion (n = 24). List of articles that did not fulfill the eligibility criteria in phase 2. (DOC 46 kb) [file 13643_2017_587_MOESM3_ESM.doc]

**Appendix II -** Excluded articles and reasons for exclusion (n=13).

| **Author, year** | **Reasons for** **exclusions** |
| --- | --- |
| Fiorini et al. (1), 2013 | 2 |
| Herrera et al. (2), 2009 | 2 |
| Jaramillo et al. (3), 2012 | 2 |
| Michalowicz et al. (4), 2009 | 2 |
| Reddy et al. (5). 2014 | 2 |
| Sant’ana et al. (6), 2011 | 2 |
| Sharma et al. (7), 2009 | 1 |
| Tarannum and Faizuddin (8), 2007 | 2 |
| Tarannum et al. (9), 2011 | 1 |
| Tarannum and Faizuddin (10), 2012 | 1 |
| Tian et al. (11). 2015 | 2 |
| Weidlich (12), 2009 | 2 |
| Yalcin et al. (13), 2002 | 1 |

1 - Cross-sectional, Case-control, Cohort studies or any interventional design other than randomized clinical trials; 2 - Studies which no primary outcome (preterm birth, low birth weight or pre-eclampsia) or in which biomarkers were not tested.

REFERENCES

1. Fiorini, T., Susin, C., da Rocha, J.M., Weidlich, P., Vianna, P., Moreira, C.H., Bogo Chies, J.A., Rösing, C.K., Oppermann, R.V., 2013. Effect of nonsurgical periodontal therapy on serum and gingival crevicular fluid cytokine levels during pregnancy and postpartum. J. Periodont. Res. 48,126-133.
2. Herrera, J.A., Velez-Medina, S., Molano, R.M., Medina, V., Botero, J.E., Parra, B., Contreras A., 2009. Periodontal intervention effects on pregnancy outcomes in women with preeclampsia. Colombia medica 40,177-184.
3. Jaramillo, A., Arce, R., Contreras, A., Herrera, J.A., 2012. Efecto del tratamiento periodontal sobre la microbiota subgingival en pacientes con preeclampsia. Biomédica 32,233-238.
4. Michalowicz, B.S., Novak, M.J., Hodges, J.S., DiAngelis, A., Buchanan, W., Papapanou, P.N.,  Mitchell, D.A., Ferguson, J.E., Lupo, V., Bofill, J., Matseoane, S., Steffen, M., Ebersole, J.L., 2009. Serum Inflammatory Mediators in Pregnancy: Changes After Periodontal Treatment and Association With Pregnancy Outcomes. J. Periodontol. 80,1731-1741.
5. Reddy, B.V.R., Tanneeru, S., Chava, V.K., 2014. The effect of phase-I periodontal therapy on pregnancy outcome in chronic periodontitis patients. J. Obstet. Gynaecol. 34,29-32.
6. Sant'ana, A.C.P., Campos, M.R., Passanezi, S.C., Rezende, M.L.R., Greghi, S.L.A., Passanezi, E., 2011. Periodontal treatment during pregnancy decreases the rate of adverse pregnancy outcome: a controlled clinical trial. J. Appl. Oral. Sci. 19,130-136.
7. Sharma, A., Ramesh, A., Thomas, B., 2009. Evaluation of plasma C-reactive protein levels in pregnant women with and without periodontal disease: A comparative study. J. Indian. Soc. Periodontol. 13,145-149.
8. Tarannum, F., Faizuddin, M., 2007. Effect of periodontal therapy on pregnancy outcome in women affected by periodontitis. J. Periodontol. 78,2095-2103.
9. Tarannum, F., Faizuddin, M., Madaiah, H., 2011. Gingival crevicular fluid prostaglandin E2 level as a predictor of preterm low birth weight: a pilot investigation. J. Oral. Sci. 53,293-300.
10. Tarannum, F., Faizuddin, M., 2012. Association between gingival crevicular fluid prostaglandin E2 level and preterm low birth weight. Indian. J. Dent. Res. 23,120.
11. Tian, Y.G., Qiu, L.H., Chen, X.B., Deng, N., Hao, C.B., 2015. Effect of nonsurgical periodontal treatment on C – Reactive protein level in pregnant women with periodontitis. J. Dalian. Med. Univ. 37,142-144.
12. Weidlich, P., Doenças periodontais e desfechos gestacionais adversos. Porto Alegre. [Tese de Doutorado em Odontologia] Universidade Federal do Rio Grande do Sul. 2009.
13. Yalcin, F., [Basegmez, C](http://www.ncbi.nlm.nih.gov/pubmed/?term=Basegmez C%5BAuthor%5D&cauthor=true&cauthor_uid=11895282)., [Isik, G](http://www.ncbi.nlm.nih.gov/pubmed/?term=Isik G%5BAuthor%5D&cauthor=true&cauthor_uid=11895282)., [Berber, L](http://www.ncbi.nlm.nih.gov/pubmed/?term=Berber L%5BAuthor%5D&cauthor=true&cauthor_uid=11895282)., [Eskinazi, E](http://www.ncbi.nlm.nih.gov/pubmed/?term=Eskinazi E%5BAuthor%5D&cauthor=true&cauthor_uid=11895282)., Soydinc, M., Issever, H., Onan, U., 2002. The effects of periodontal therapy on intracrevicular prostaglandin E2 concentrations and clinical parameters in pregnancy. J. Periodontol. 73,173-177.
