# Supplementary material for: Effect of intra-pregnancy nonsurgical periodontal therapy on inflammatory biomarkers and adverse pregnancy outcomes: a systematic review with meta-analysis
Source: Syst Rev. 2017 Oct 10;6:197. doi: 10.1186/s13643-017-0587-3 (PMC5635531; doi:10.1186/s13643-017-0587-3)
Supplement: Supplementary file 4 — Appendix III presents the risk of bias in select papers.Risk of bias assessed by The Cochrane Collaboration’s tool for assessing risk of bias. Assessment of bias risk in the selected studies in the search strategy. (DOC 33 kb) [file 13643_2017_587_MOESM4_ESM.doc]

**Appendix III -** Risk of bias assessed by The Cochrane Collaboration’s tool for assessing risk of bias^1^.

|  | Offenbacher et al., 2006 | Khairnar et al., 2015 | Penova-Veselinovic et al., 2015 | Pirie et al., 2013 |
| --- | --- | --- | --- | --- |
| Was the allocation sequence adequately generated? | UU | UU | UU | YY |
| Was allocation adequately concealed? | UU | UU | UU | YY |
| Was knowledge of the allocated interventions adequately prevented during the study? | UU | UU | UU | UU |
| Were incomplete outcome data adequately addressed? | YY | UU | YY | YY |
| Are reports of the study free of suggestion of selective outcome reporting? | YY | NN | YY | YY |
| Was the study apparently free of other problems that could put it at a risk of bias? | YY | NN | YY | YY |

1. Higgins JPT, Green S (editors). *Cochrane Handbook for Systematic Reviews of Interventions* Version 5.1.0 [updated March 2011]. The Cochrane Collaboration, 2011. Available from [www.cochrane-handbook.org](http://www.cochrane-handbook.org/).

Y – Yes; N – No; U – Unclear
